# Fiberoptic bronchoscopy for the rapid diagnosis of smear-negative pulmonary tuberculosis

**DOI:** 10.1186/1471-2334-12-141

**Published:** 2012-06-22

**Authors:** Jung Ar Shin, Yoon Soo Chang, Tae Hoon Kim, Hyung Jung Kim, Chul Min Ahn, Min Kwang Byun

**Affiliations:** 1 Department of Internal Medicine, Yonsei University College of Medicine, Gangnam Severance Hospital, 211 Eonju-ro, Gangnam-gu Seoul, 135-720, South Korea; 2 Department of Radiology, Yonsei University College of Medicine, Gangnam Severance Hospital, 211 Eonju-ro, Gangnam-gu, 211 Eonju-ro, Gangnam-gu, Seoul 135-720 135-720, , South Korea

## Abstract

**Background:**

This study was aimed to investigate the diagnostic value of fiberoptic bronchoscopy (FOB) with chest high-resolution computed tomography (HRCT) for the rapid diagnosis of active pulmonary tuberculosis (PTB) in patients suspected of PTB but found to have a negative sputum acid-fast bacilli (AFB) smear.

**Methods:**

We evaluated the diagnostic accuracy of results from FOB and HRCT in 126 patients at Gangnam Severance Hospital (Seoul, Korea) who were suspected of having PTB.

**Results:**

Of 126 patients who had negative sputum AFB smears but were suspected of having PTB, 54 patients were confirmed as having active PTB. Hemoptysis was negatively correlated with active PTB. Tree-in-bud appearance on HRCT was significantly associated with active PTB. The sensitivity, specificity, positive predictive value (PPV), and negative predictive value (NPV) of FOB alone was 75.9%, 97.2%, 95.3%, and 84.3%, respectively, for the rapid diagnosis of active PTB. The combination of FOB and HRCT improved the sensitivity to 96.3% and the NPV to 96.2%.

**Conclusions:**

FOB is a useful tool in the rapid diagnosis of active PTB with a high sensitivity, specificity, PPV and NPV in sputum smear-negative PTB-suspected patients. HRCT improves the sensitivity of FOB when used in combination with FOB in sputum smear-negative patients suspected of having PTB.

## Background

Tuberculosis (TB) is a major health problem around the world, with an approximate incidence of 9.4 million (8.9–9.9 million) and with about 1.7 million TB-related deaths in 2009 [1,2]. The global strategy to control TB is prompt diagnosis, notification, and successful treatment of patients with active, transmissible disease. Early diagnosis of active pulmonary tuberculosis (PTB) is critical for TB control.

Unfortunately, diagnosis of active PTB is often delayed because fewer than half of these patients have a positive sputum smear (29–39% in 2009 in Korea) [1,3], and isolation of *Mycobacterium tuberculosis* (MTB) takes a long time. Acid-fast bacilli (AFB) smears of respiratory specimens (at least two or more specimens) are important for the prompt diagnosis of PTB, but AFB smears have poor sensitivity (30–70%) despite high specificity (98–99%). Mycobacterial cultures are more sensitive than AFB smears (80–85%), but culture results usually require 3–8 weeks [4]. The diagnosis of TB and the decision to start treatment against sputum smear-negative TB is usually dependent on clinical features, but 20% of PTB patients are completely asymptomatic whereas 42–86% of PTB patients may be symptomatic. Sputum smear-negative PTB patients are especially likely to show no or mild respiratory symptoms and systemic manifestations [5].

Chest x-ray is another method often used in PTB screening, but in sputum smear-negative TB, many cases showed atypical x-ray patterns or normal findings [6]. In addition, this method has the limitation of a high false positive rate due to previous PTB infection in an intermediate or high TB burden country. To assist in the diagnosis of sputum smear-negative PTB and latent TB infection (LTBI), clinicians use the tuberculin skin test (TST). However, the TST is limited in application due to cross-reactivity with non-tuberculous mycobacteria (NTM) species and Bacillus Calmette-Geurin (BCG) vaccine strains, and its sensitivity is affected by both malnutrition and immunosuppression [7].

Chest high-resolution computed tomography (HRCT) provides information about the extent and distribution of PTB and can be a great help in identifying its activity. In sputum smear-negative cases, HRCT is superior to chest x-ray in the diagnosis of PTB and in the determination of its extent and distribution. Further, it confirms the presumptive diagnosis of active PTB and allows anti-tuberculosis treatment to commence more quickly. Despite some disadvantages such as high cost and radiation exposure, HRCT is widely used when traditional methods have failed to diagnose PTB in sputum smear-negative patients. Typical radiological patterns of PTB reactivation such as upper lobe involvement or cavity formation are rarely observed in sputum smear-negative cases due to the smaller burden of mycobacterium [5]; therefore, HRCT alone is limited in diagnosing PTB in sputum smear-negative patients.

Fiberoptic bronchoscopy (FOB) can provide alternative respiratory specimens for diagnosis [8,9], especially from specific sites that are suspected by radiological testing for involvement of PTB when sputum expectoration has repeatedly failed because sputum is absent [10]. FOB is also more useful in the diagnosis of endobronchial TB, which can be seen as normal in HRCT, and FOB may be superior in the differential diagnosis of tuberculosis with other commonly encountered diseases such as pneumonia or lung cancer [11]. Despite the fact that it is more aggressive and relatively expensive, FOB is considered useful for the diagnosis of sputum smear-negative PTB because of these advantages. Results of bronchial washing specimens with AFB smears or MTB polymerase chain reaction (PCR) and tissue specimens from bronchoscopic or transbronchial biopsies are usually received within one week, thus enabling rapid diagnosis before the availability of confirmation from sputum cultures of sputum smear-negative PTB patients.

The aim of this study was to evaluate the diagnostic value of FOB with HRCT in the diagnosis of active PTB in patients suspected of PTB but presenting with a negative sputum smear in an intermediate TB burden country.

## Methods

### Study setting and subjects

We retrospectively reviewed the clinical records and FOB and HRCT results of all patients with suspected PTB who visited the pulmonary clinic of Gangnam Severance Hospital, Seoul, Korea from January 2009 to December 2010.

Inclusion criteria were (1) age of at least 18 years with clinical or radiographic suspicion of active PTB, (2) negative AFB smear results of 2 or more sputum pairs or failure to expectorate sputum (in other words, could not expectorate sputum voluntarily, did not submit any sputum specimen) and (3) having received both FOB and HRCT within 1 month due to suspected PTB before initiation of anti-TB treatment. When FOB was performed, a bronchial washing from the affected lung was acquired for AFB smear, mycobacterial culture and MTB-PCR for all patients. A bronchoscopic biopsy was performed on patients with an endobronchial lesion. Exclusion criteria were (1) AFB smear-positive patients, (2) patients with clinically diagnosed PTB according to clinical and radiological tuberculosis findings who showed no clinical improvement with empirical antibiotics but did show clinical and radiological improvement with anti-tuberculosis medication, and (3) patients with inconclusive diagnoses due to loss of follow-up.

From January 2009 to December 2010, 182 patients who were suspected of having PTB visited our clinic and underwent FOB and chest CT. HRCT was performed with contrast CT, simultaneously. After excluding smear-positive PTB patients and patients with other exclusion criteria, we evaluated the diagnostic accuracy of FOB and HRCT results in 126 patients. The recorded clinical information on these patients included age, gender, cough, sputum, fever, hemoptysis, chest pain, and weight loss.

Bronchoscopic procedure is performed according to our institute’s infection regulation and instruction guideline. Inspectors wear N95 masks, goggles and gown during procedure, bronchosopy room is equipped with negative pressure isolation and air disinfection system, Bronchoscopy is performed according to manufacturer’s guidelines plus scope surveillance.

### Diagnostic definition

Active PTB was confirmed when (1) MTB was cultured or (2) a caseating granuloma was found in the lung tissue by bronchoscopic biopsy or transthoracic needle biopsy, and when the PTB showed appropriate response to treatment. In cases diagnosed by pathology, active PTB was confirmed only if MTB tissue culture or tissue MTB-PCR was positive, for excluding NTM or other granulomatous diseases. A final diagnosis of ‘non-TB’ was accepted when an alternative diagnosis was reached.

A rapid diagnosis of sputum smear-negative PTB by FOB was defined as a diagnosis of PTB through methods that yielded results within 1 week: (1) a positive AFB smear, (2) positive MTB-PCR, or (3) a caseating granuloma upon biopsy.

CT findings were reviewed by two experienced thoracic radiologists, without clinical information or final diagnosis in each case. CT findings were described as having more than one of the following characteristics: (1) consolidation, (2) cavities, (3) centrilobular air space nodules, (4) tree-in-bud appearance and (5) hilar and/or mediastinal lymph node enlargement. After reviewing the CT findings independently, the two radiologists met, discussed and reached a final diagnosis by consensus.

The definition of an immunocompromised condition included the following: (1) having a diagnosis of diabetes mellitus, (2) undergoing chemotherapy for an underlying malignancy at the time of TST and QuantiFERON®-TB Gold In-Tube (QFT-IT, Cellestis Ltd; Carnegie, Australia) testing, (3) having had received either a solid organ transplant or bone marrow transplant, (4) having a diagnosis of end-stage renal disease and on renal replacement therapy, (5) having a diagnosis of advanced liver cirrhosis with Child-Pugh class C, (6) being seropositive for human immunodeficiency virus, or (7) undergoing daily administration of systemic corticosteroids (at least 15 mg prednisone per day for more than 1 month) or combination therapy with low dose corticosteroids and other immunosuppressants including azathioprine, mycophenolate, methotrexate, cyclosporine, or cyclophosphamide.

For the combination of FOB and HRCT, the results of the combination were considered positive when at least one test was positive.

### Analysis

Data were analyzed with SPSS statistical software (version 18.0; SPSS; Chicago, IL, USA). Univariate comparisons between active PTB and non-TB patients were performed using Fisher’s exact test for categorical variables and the Mann–Whitney test for continuous variables, where appropriate. Associations of clinical and radiological parameters with active PTB diagnosis were analyzed using univariate or multivariate logistic regression modeling. For multivariate analysis, variables were incorporated into the model in a stepwise manner. All tests of significance were two-sided and a P value < 0.05 was considered statistically significant. Odds ratios (ORs) and 95% confidence intervals (95% CIs) were calculated. Sensitivity, specificity, positive predictive value (PPV) and negative predictive value (NPV) for the diagnosis of active PTB disease were calculated for each diagnostic test.

### Ethical issues

This study was approved by the Institutional Review Board of the Gangnam Severance Hospital, Yonsei University College of Medicine (IRB No: 3-2011-0118).

## Results

### Demographic characteristics

**Figure 1:**
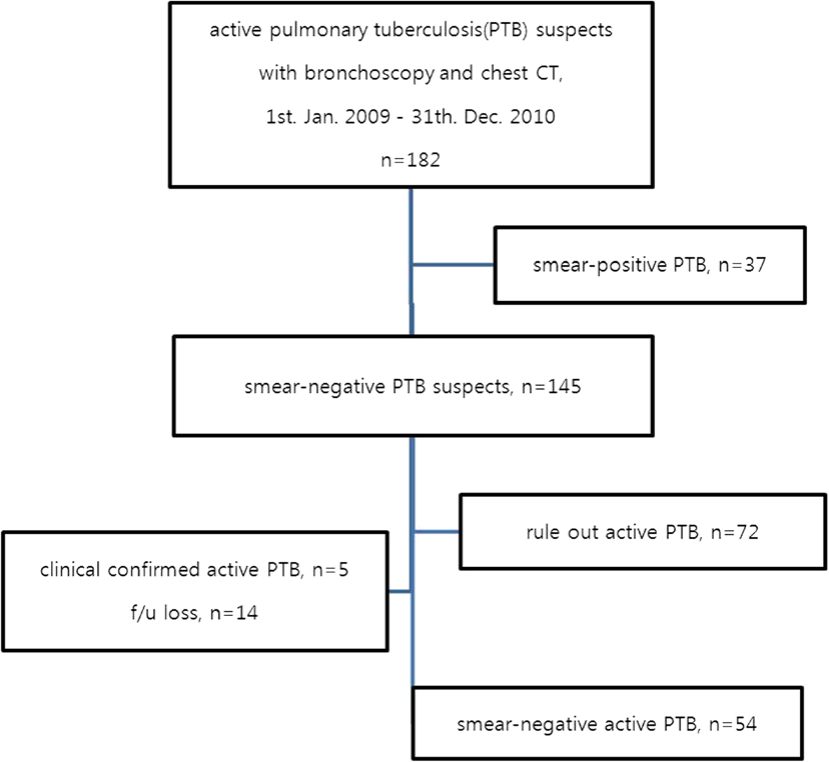
**Study flowchart.***Definition of abbreviations: FOB* = fiberoptic bronchoscopy, *CT* = computed tomography, *N* = number, *TB* = tuberculosis.

Of the 145 patients with suspected sputum smear-negative TB, five patients were excluded due to clinically diagnosed PTB, and 14 patients were excluded due to inconclusive diagnoses (Figure 1).

Baseline characteristics of the non-excluded 126 patients are summarized in Table 1. Active PTB was diagnosed in 54 patients (42.9%), of which 48 (88.9%) had positive TB cultures and 6 (11.1%) cases were confirmed by pathology and MTB-PCR. Of 48 culture-confirmed cases, 28 were culture positive by both sputum and bronchial washing specimens, 6 by sputum only, and 14 by bronchial washing specimens only.

**Table 1:** Demographic and clinical characteristics of all sputum smear-negative TB suspects

**Characteristic**	**All suspects**	**TB**	**Non-TB**	**P-value**
	**N = 126**	**N = 54**	**N = 72**	
Age, years (mean ± SD)	51.0 ± 18.2	45.3 ± 18.8	55.4 ± 16.6	0.002
Male, n (%)	68 (54.0)	26 (48.1)	42 (58.3)	0.256
Previous TB history, n (%)	33 (26.2)	8 (14.8)	25 (34.7)	0.014
Immunocompromised patient, n (%)	11 (8.7)	4 (7.4)	7 (9.7)	0.649
CXR positivity, n (%)	44 (34.9)	29 (53.7)	15 (20.8)	< 0.001
QFT-IT positivity^*^, n (%)	33/51 (64.7)	20/23 (87.0)	13/28 (46.4)	0.009
Active pulmonary TB, n (%)	54 (42.9)	54 (100.0)		
Culture confirmed, n (%)^†^		48 (88.9)		
Biopsy confirmed, n (%)		6 (11.1)		

*Definition of abbreviations: TB* = tuberculosis, *SD* = standard deviation, *n* = number, *CXR* = chest x-ray, *QFT-IT* = QuantiFERON®-TB Gold In-Tube.

*51 of 126 cases were examined with this test.

^†^Of the 48 culture-confirmed cases, 28 showed cultures positive in both sputum and bronchial washing specimens, 6 only in sputum and 14 only in bronchial washing specimens.

Alternative (non-TB) diagnoses were made for 72 patients (34 pneumonia, 5 NTM colonization, 15 sequelae of a previous TB infection, 12 lung cancers, and 6 interstitial lung disease).

The mean patient age was 51.0 ± 18.2 years, and the mean age of the non-TB group was higher than that of the TB group (55.4 ± 16.6 years vs. 45.3 ± 18.8 years, respectively, P = 0.002). Immunocompromised conditions were found in 11 patients (6 diabetes, 2 chemotherapy, 2 hematologic malignancies, and 1 systemic corticosteroid use). There was no difference between the TB and non-TB groups in terms of immunocompromised status, but previous TB history was significantly different between the TB and non-TB groups (P = 0.014, Table 1).

### Clinical characteristics and HRCT findings in sputum smear-negative TB suspects

Chest x-rays showed a higher positive rate in the TB group than in the non-TB group (53.7% vs. 20.8%, respectively, P < 0.001), and QFT-IT also showed significantly higher positive rate in the TB group than in the non-TB group (87.0% vs. 46.4%, respectively, P = 0.009) (Table 1). Only hemoptysis (P = 0.009, OR = 0.30 [0.12–0.76, 95% CI]) was negatively correlated with active PTB in sputum smear-negative suspects (Table 2).

**Table 2:** First presenting symptoms of patients with sputum smear-negative TB

**Symptoms**	**All suspects**	**TB**	**Non-TB**	**P-value**	**OR (95% CI)**
	**N = 126**	**N = 54**	**N = 72**		
Cough, n (%)	70 (55.6)	34 (63.0)	36 (50.0)	0.147	1.70 (0.827–3.493)
Sputum, n (%)	42 (33.3)	18 (33.3)	24 (33.3)	1.000	1.00 (0.473–2.114)
Fever, n (%)	27 (21.4)	13 (24.1)	14 (19.4)	0.531	1.31 (0.559–3.087)
Hemoptysis, n (%)	31 (24.6)	7 (13.0)	24 (33.3)	0.009	0.30 (0.117–0.757)
Chest wall pain, n (%)	22 (17.5)	13 (24.1)	9 (12.5)	0.090	2.22 (0.870–5.662)
Weight loss, n (%)	4 (3.2)	3 (5.6)	1 (1.4)	0.313	4.18 (0.422–41.308)

*Definition of abbreviations: TB* = tuberculosis, *n* = number, *OR* = odds ratio, 95% *CI* = 95% confidence interval.

Fifty-three patients were suspected of having active PTB by HRCT, and 38 (71.7%) were correctly diagnosed with active PTB. Of the 73 patients suspected to be non-TB, 58 (79.5%) were correctly excluded. HRCT findings in the diagnosis of PTB showed a significant difference between TB and non-TB patients (P < 0.001) (data not shown). The most frequently observed CT findings (in order of decreasing frequency) were consolidation, mediastinal lymphadenopathy, tree-in-bud appearance, and nodules. Tree-in-bud nappearance was the only significant finding associated with active PTB status (P < 0.001, OR = 5.10 [2.37–10.99, 95% CI]) (Table 3).

**Table 3:** Multivariate regression analysis of CT findings in sputum smear-negative TB suspects

**Characteristic**	**All suspects**	**TB**	**Non-TB**	**P-value**	**OR (95% CI)**
	**N = 126**	**N = 54**	**N = 72**		
Consolidation, n (%)	88 (69.8)	38 (70.4)	50 (69.4)	0.726	1.05 (0.484–2.257)
Nodule, n (%)	50 (39.7)	23 (42.6)	27 (37.5)	0.435	1.24 (0.602–2.541)
Cavity, n (%)	23 (18.3)	15 (27.8)	8 (11.1)	0.050	3.08 (1.195–7.924)
Tree-in-bud, n (%)	52 (41.3)	34 (63.0)	18 (25.0)	<0.001	5.10 (2.366–10.991)
Mediastinal LAP, n (%)	53 (42.1)	26 (48.1)	27 (37.5)	0.940	1.55 (0.756–3.167)

*Definition of abbreviations: CT* = computed tomography, *TB* = tuberculosis, *n* = number, *OR* = odds ratio, 95% *CI* = 95% confidence interval, *LAP* = lymphadenopathy.

### Bronchoscopic characteristics in PTB patients

Of the 54 patients confirmed with active PTB, 41 patients had TB and 13 patients did not have TB according to rapid diagnosis via bronchoscopic methods. As a result, two non-TB patients were misdiagnosed as having TB by bronchoscopic rapid diagnosis ( Additional file 1: Table S1). Baseline characteristics were not different between TB and non-TB patients divided by bronchoscopic diagnosis ( Additional file 1: Table S2).

The bronchoscopic findings are summarized in Table 4. Of the 54 PTB-confirmed patients, 42 patients had positive bronchial washing cultures (Table 4), 34 patients had positive sputum cultures, and 28 patients showed a positive result on both the sputum and bronchial washing cultures, but 14 patients had positive only in bronchial washing cultures ( Additional file 1: Table S3).

**Table 4:** Bronchoscopic findings in 54 patients confirmed to have active PTB

	**TB**	**Culture confirmed**	**Biopsy confirmed**
	**N = 54**	**N = 48**	**N = 6**
Bronchial washing culture	42/54 (77.8)	42/48 (87.5)	0/6 (0.0)
Bronchial washing AFB smear	14/54 (25.9)	12/48 (25.0)	2/6 (33.3)
Bronchial washing MTB-PCR	33/54 (61.1)	32/48 (66.7)	1/6 (16.7)
Bronchoscopic biopsy (TBB + bronchial biopsy)	22/23 (95.7)	16/17 (94.1)	6/6 (100.0)
Bronchial washing AFB smear + MTB-PCR + bronchoscopic biopsy	41/54 (75.9)	35/48 (72.9)	6/6 (100.0)

*Definition of abbreviations: PTB* = pulmonary tuberculosis, *TB* = tuberculosis, *AFB* = acid-fast bacilli, *MTB* = mycobacterial tuberculosis, *PCR* = polymerase chain. reaction, *TBB* = transbronchial lung biopsy.

^*^Numbers of patients with positive results in each test/total numbers of patients who underwent each test (%).

Of the 54 PTB-confirmed patients, 41 (75.9%) patients were rapidly diagnosed in less than 1 week by bronchial washing AFB smear, MTB-PCR and/or biopsy, and 13 patients were confirmed several weeks later by bronchial washing or sputum culture results. AFB smears produced by bronchial washing showed positive results for 25.9% (14/54) of the PTB-confirmed patients, and bronchoscopic biopsy results were positive in 22 of 23 patients who underwent a biopsy (Table 4). Of 23 patients who underwent a biopsy, a transbronchial biopsy was performed in 5 patients and a bronchial biopsy was performed in 18 patients with an endobronchial lesion (data not shown). MTB-PCR from a bronchial washing showed positive results in 61.1% (33/54) of patients (Table 4), and 2 of the finally diagnosed 72 non-TB patients showed false-positive results ( Additional file 1: Table S1).

### Diagnostic accuracy of FOB and HRCT in sputum smear-negative TB suspects

The diagnostic accuracy of FOB, HRCT, and the combination of FOB and HRCT in sputum smear-negative TB suspects is summarized in Table 5. The sensitivity of FOB in the rapid diagnosis of active PTB was 75.9% (95% CI, 69.0–78.6%), and the specificity was 97.2% (95% CI, 92.0–99.2%). The sensitivity of HRCT in the diagnosis of active PTB was 85.2% (95% CI, 75.2–92.3%), and the specificity was 72.2% (95% CI, 64.7–77.6%). The PPV of FOB was 95.3% (95% CI, 86.7–98.7%) and the NPV of FOB was 84.3% (95% CI, 79.8–86.1%) in the rapid diagnosis of active PTB. These values were higher than the PPV of HRCT (69.7% [95% CI, 61.5–75.5%]) and similar to the NPV of HRCT (86.7% [95% CI, 77.7–93.1%]). The combination of FOB and HRCT improved the sensitivity to 96.3% and the NPV to 96.2%.

**Table 5:** Diagnostic accuracy in all sputum smear-negative TB suspects

	**Sensitivity (95% CI)**	**Specificity (95% CI)**	**PPV (95% CI)**	**NPV (95% CI)**	**+LR (95% CI)**	**−LR (95% CI)**
FOB	0.759 (0.690–0.786)	0.972 (0.920–0.992)	0.953 (0.867–0.987)	0.843 (0.798–0.861)	27.333 (8.674–99.257)	0.248 (0.216–0.337)
CT	0.852 (0.752–0.923)	0.722 (0.647–0.776)	0.697 (0.615–0.755)	0.867 (0.777–0.931)	3.067 (2.131–4.117)	0.205 (0.099–0.383)
FOB + CT	0.963 (0.879–0.993)	0.708 (0.645–0.731)	0.712 (0.650–0.735)	0.962 (0.876–0.993)	3.302 (2.477–3.696)	0.052 (0.009–0.188)

*Definition of abbreviations*: *TB* = tuberculosis, *PPV* = positive predictive value, *NPV* = negative predictive value, +*LR* = positive likelihood ratio, -*LR* = negative likelihood ratio, 95% *CI* = 95% confidence interval, *FOB* = fiberoptic bronchoscopy, *CT* = computed tomography.

## Discussion

In this retrospective study, we found that FOB showed high sensitivity, specificity, PPV, and NPV in the rapid diagnosis of sputum smear-negative PTB, and the combination of FOB and HRCT increased the sensitivity and NPV.

Several previous studies evaluated clinical characteristics and scoring systems for the diagnosis of sputum smear-negative PTB [12-14]. Samb et al. [12] reported independent predictors of active PTB including a chronic cough lasting longer than 3 weeks, chest pains longer than 15 days, absence of sputum, and absence of dyspnea, and Lee et al. [14] reported that the lack of sputum was a positive predictor of active PTB. But even in these studies, the specificity of the clinical predictors or scoring system was low, and the PPV was reported at a mere 50%. In our study, hemoptysis was the only negative predictor in sputum smear-negative TB suspects, and other clinical characteristics did not help in the prediction of active PTB.

We discovered that tree-in-bud appearance was a meaningful HRCT finding in the diagnosis of sputum smear-negative PTB. Recently, several studies have reported that tree-in-bud appearance, lobular consolidation and large nodules were positively associated with active PTB in sputum smear-negative TB suspects, but the diagnostic accuracy was not satisfactory [14,15]. Matsuoka et al. [16] stated that CT findings in sputum smear-negative patients differed from those in smear-positive patients and suggested that CT findings are not helpful in judging sputum smear-negative TB suspects.

Despite being less infectious than sputum smear-positive PTB, smear-negative PTB serves as an important cause of transmission in communities by delaying diagnosis and precluding initiation of treatment and often leads to complications of irreversible lung damage in infected individuals [4]. Therefore, sputum smear-negative PTB often requires more invasive diagnostic tools to be distinguished from other diseases such as lung cancer. In addition, in intermediate or high TB burden countries such as South Korea, beginning unnecessary anti-TB treatment prior to receiving results of mycobacterial culture may cause unnecessary economic burden to the community and drug side effects to the patient.

Lee et al. [14] reported that the sensitivity and the specificity of sputum TB-PCR was 43.2% (95% CI, 27–60%) and 97.7% (95% CI, 86–99%), respectively. Because of its low sensitivity and NPV (66.7% [95% CI, 54–78%]), sputum TB-PCR alone was limited in ruling out TB. In a recent meta-analysis study [17], the sensitivity and the specificity of QFT-IT was 80% (95% CI, 75–84%) and 79% (95% CI, 78–84%), respectively, and the sensitivity and the specificity of T-SPOT TB was 81% (95% CI, 78–84%) and 59% (95% CI, 56–62%), respectively. The diagnostic sensitivity of both interferon-gamma release assays (IGRAs) was higher than that of the TST, but the sensitivity of IGRAs was still not high enough for IGRAs to be used as a rule out test for TB. In addition, the specificity of the IGRAs was not sufficient for the diagnosis of active TB disease with differentiation from latent TB infection.

In previous studies, FOB had a sensitivity of 80–93% and a specificity of 70–95% [8,9,18,19] for rapid diagnosis of sputum smear-negative PTB, and HRCT had a sensitivity of 60–80% and a specificity of 50–70% [14,20,21]. Our results indicate that HRCT has a similar sensitivity of 85.2% and specificity of 72.2%, but HRCT alone is limited as usual in the diagnosis of PTB in sputum smear-negative TB suspects due to its low PPV of 69.7% (positive likelihood ratio (LR+), 3.067 [95% CI, 2.131–4.117]). We found that FOB alone is useful for the rapid diagnosis of sputum smear-negative PTB with a high PPV of 95.3% (LR + = 27.333 [95% CI, 8.674–99.257]). FOB is also useful for the exclusion of non-TB cases from patients suspected of having active PTB with a high specificity and NPV in our setting. The combination of FOB with HRCT increased the sensitivity to 96.3% and NPV to 96.2%.

The high cost and concerns about the invasiveness of FOB and radiation exposure of HRCT limit the usefulness of these tests, but FOB is a safe and widely performed procedure. Previous studies [22,23] have reported that with the help of oxygenation, adequate premedication, and performance by experienced physicians, FOB shows very low complication rates and few life-threatening side effects. One previous study investigating the cost-effectiveness of FOB and HRCT in the diagnosis of PTB [21] reported that FOB and HRCT play significant roles in the moderate or high PTB probability setting compared with the low PTB probability setting. Considering the high specificity of our study, FOB and/or HRCT would be useful in a high TB burden country as well as in an intermediate TB burden country such as South Korea.

There are several limitations in this study. First, it was a retrospective study and the study population and clinical setting were selective and limited, so it is difficult to generalize this result to other settings. Second, the commonly used TST was not evaluated in our study. This was based on previous studies showing the limitation of TST for evaluating TB in South Korea due to BCG vaccination [24,25]. Third, the use of FOB in the diagnosis of PTB is not clinically available worldwide. To improve diagnostic accuracy and ensure safety, a well-trained pulmonologist is essential. High cost and the complication risk limit the use of FOB in other situations. However, our study demonstrates that FOB and FOB with HRCT can play an important role in the rapid diagnosis of active PTB in sputum smear-negative patients suspected of TB.

## Conclusions

In conclusion, our results suggest that FOB has good sensitivity, specificity, PPV, and NPV and was useful in the rapid diagnosis of sputum smear-negative PTB and the exclusion of non-TB in sputum smear-negative TB-suspected patients. HRCT alone was limited for the diagnosis of active PTB, but the combination of FOB and HRCT may improve the sensitivity of FOB in the rapid diagnosis of sputum smear-negative PTB. Based on our results, we suggest that physicians actively consider performing FOB and HRCT in sputum smear-negative patients suspected of TB.

## Abbreviations

AFB: Acid-fast bacilli; BCG: Bacillus Calmette-Geurin; FOB: Fiberoptic bronchoscopy; HRCT: High-resolution computed tomography; IGRAs: Interferon-gamma release assays; LTBI: Latent TB infection; MTB: *Mycobacterium tuberculosis*; NPV: Negative predictive value; NTM: Non-tuberculous mycobacteria; PCR: Polymerase chain reaction; PPV: Positive predictive value; PTB: Pulmonary tuberculosis; QFT-IT: QuantiFERON®-TB Gold In-Tube; TB: Tuberculosis; TST: Tuberculin skin test.

## Competing interests

The author(s) declare that they have no competing interests.

## Authors’ contributions

All authors have read and approved the final manuscript. SJA and BMK designed and conducted the study; collected, analyzed, and interpreted the data; and wrote the manuscript. KTH, CYS, KHJ, and ACM analyzed and interpreted the data and contributed to the writing of the manuscript. All authors read and approved the final manuscript.

## Pre-publication history

The pre-publication history for this paper can be accessed here:

http://www.biomedcentral.com/1471-2334/12/141/prepub

## Supplementary Material

Additional file 1 **Tables S1-S3.** Bronchoscopic findings of all subjects. Tables S2. Diagnostic and clinical characteristics of active PTB confirmed 54 patients according to bronchoscopic diagnosis. Tables S3. Bronchoscopic findings in 54 active PTB patients according to sputum mycobacteria culture results. Click here for file
