# Supplementary material for: Fiberoptic bronchoscopy for the rapid diagnosis of smear-negative pulmonary tuberculosis
Source: BMC Infect Dis. 2012 Jun 22;12:141. doi: 10.1186/1471-2334-12-141 (PMC3507815; doi:10.1186/1471-2334-12-141)
Supplement: Additional file 1 — Tables S1-S3. Bronchoscopic findings of all subjects. Tables S2. Diagnostic and clinical characteristics of active PTB confirmed 54 patients according to bronchoscopic diagnosis. Tables S3. Bronchoscopic findings in 54 active PTB patients according to sputum mycobacteria culture results. [file 1471-2334-12-141-S1.doc]

**Supplementary Table 1** Bronchoscopic findings of all subjects^*^

|  | TB  N = 54 | Non-TB  N = 72 |
| --- | --- | --- |
| Bronchial washing culture | 42/54 (77.8) | 0/72 (0.0) |
| Bronchial washing AFB smear | 14/54 (25.9) | 0/72 (0.0) |
| Bronchial washing MTB-PCR | 33/54 (61.1) | 2/72 (2.8) |
| Bronchoscopic biopsy (TBB + bronchial biopsy) | 22/23 (95.7) | 0/20 (0.0) |
| Bronchial washing AFB smear + MTB-PCR + bronchoscopic biopsy | 41/54 (75.9) | 2/72 (2.8) |
| *Definition of abbreviations:* PTB = pulmonary tuberculosis, TB = tuberculosis, AFB = acid-fast bacilli, MTB = mycobacterial tuberculosis, PCR = polymerase chain reaction, TBB = transbronchial lung biopsy  ^*^ Numbers of patients with positive in each test/total numbers of patients underwent each test (%) | | |

**Supplementary Table 2** Diagnostic and clinical characteristics of active PTB confirmed 54 patients according to bronchoscopic diagnosis

| Characteristic | TB  N = 54 | Bronchoscopic rapid diagnosis | | P-value |
| --- | --- | --- | --- | --- |
|  |  | Positive | Negative |  |
|  |  | N = 41 | N = 13 |  |
| Age, years, mean ± SD | 45.26±18.80 | 46.95±19.57 | 39.92±15.64 | 0.279 |
| Male, n (%) | 26 (48.1) | 18 (43.9) | 8 (61.5) | 0.267 |
| Old pulmonary history, n (%) | 8 (14.8) | 7 (17.1) | 1 (7.7) | 0.407 |
| Immunocompromised pt, n (%) | 4 (7.4) | 4 (11.4) | 0 (0.0) | 0.242 |
| CXR positivity, n (%) | 29 (53.7) | 20 (48.8) | 9 (69.2) | 0.198 |
| CT positivity, n (%) | 38 (70.4) | 27 (65.9) | 11 (84.6) | 0.197 |
| QFT-IT positivity ^*^, n (%) | 20/23 (86.9) | 15/17 (88.2) | 5/6 (83.3) | 0.759 |
| *Definition of abbreviations:* TB = tuberculosis, SD = standard deviation, n = number, pt = patient, CXR = chest x-ray, QFT-IT = QuantiFERON®-TB Gold In-Tube, CT = computed tomography,  ^*^ 31 of 54 (57.4%) cases were examined with this test | | | | |

**Supplementary Table 3** Bronchoscopic findings in 54 active PTB patients according to sputum mycobacteria culture results^*^

|  | Sputum culture | | |
| --- | --- | --- | --- |
|  | Positive  N = 34 | Negative  N = 10 | No sputum  N = 10 |
|  |  |  |  |
| Bronchial washing culture | 28/34 (82.4) | 5/10 (50.0) | 9/10 (90.0) |
| Bronchial washing AFB smear | 4/34 (11.8) | 1/10 (10.0) | 9/10 (90.0) |
| Bronchial washing MTB-PCR | 24/34 (70.6) | 1/10 (10.0) | 8/10 (80.0) |
| Bronchoscopic biopsy (TBB + bronchial biopsy) | 13/14 (92.9) | 3/3 (100.0) | 6/6 (100.0) |
| Bronchial washing AFB smear + MTB-PCR + bronchoscopic biopsy | 27/34 (79.4) | 4/10 (40.0) | 10/10 (100.0) |
| *Definition of abbreviations:* PTB = pulmonary tuberculosis, AFB = acid-fast bacilli, MTB = mycobacterial tuberculosis, PCR = polymerase chain reaction, TBB = transbronchial lung biopsy  ^*^ Numbers of patients with a positive result in each test/total numbers of patients who underwent each test (%) | | | |
